# Carotid body hyperplasia and enhanced ventilatory responses to hypoxia in mice with heterozygous deficiency of PHD2

**DOI:** 10.1113/jphysiol.2012.247254

**Published:** 2013-05-20

**Authors:** Tammie Bishop, Nick P Talbot, Philip J Turner, Lynn G Nicholls, Alberto Pascual, Emma J Hodson, Gillian Douglas, James W Fielding, Thomas G Smith, Marina Demetriades, Christopher J Schofield, Peter A Robbins, Christopher W Pugh, Keith J Buckler, Peter J Ratcliffe

**Affiliations:** 1 Wellcome Trust Centre for Human Genetics, Roosevelt Drive, University of Oxford Oxford OX3 7BN, UK; 2 Department of Physiology, Anatomy and Genetics, Sherrington Building, South Parks Road University of Oxford, Oxford OX1 3PT, UK; 3 Instituto de Biomedicina de Sevilla (IBiS), Hospital Universitario Virgen del Rocio/CSIC/Universidad de Sevilla Seville, Spain; 4 Chemistry Research Laboratory, Department of Chemistry 12 Mansfield Road, University of Oxford, Oxford OX1 3TA, UK

## Abstract

Oxygen-dependent prolyl hydroxylation of hypoxia-inducible factor (HIF) by a set of closely related prolyl hydroxylase domain enzymes (PHD1, 2 and 3) regulates a range of transcriptional responses to hypoxia. This raises important questions about the role of these oxygen-sensing enzymes in integrative physiology. We investigated the effect of both genetic deficiency and pharmacological inhibition on the change in ventilation in response to acute hypoxic stimulation in mice. Mice exposed to chronic hypoxia for 7 days manifest an exaggerated hypoxic ventilatory response (HVR) (10.8 ± 0.3 *versus* 4.1 ± 0.7 ml min^−1^ g^−1^ in controls; *P* < 0.01). HVR was similarly exaggerated in *PHD2*^+/−^ animals compared to littermate controls (8.4 ± 0.7 *versus* 5.0 ± 0.8 ml min^−1^ g^−1^; *P* < 0.01). Carotid body volume increased (0.0025 ± 0.00017 in *PHD2*^+/−^ animals *versus* 0.0015 ± 0.00019 mm^3^ in controls; *P* < 0.01). In contrast, HVR in *PHD1*^−/−^ and *PHD3^−/−^* mice was similar to littermate controls. Acute exposure to a small molecule PHD inhibitor (PHI) (2-(1-chloro-4-hydroxyisoquinoline-3-carboxamido) acetic acid) did not mimic the ventilatory response to hypoxia. Further, 7 day administration of the PHI induced only modest increases in HVR and carotid body cell proliferation, despite marked stimulation of erythropoiesis. This was in contrast with chronic hypoxia, which elicited both exaggerated HVR and cellular proliferation. The findings demonstrate that PHD enzymes modulate ventilatory sensitivity to hypoxia and identify PHD2 as the most important enzyme in this response. They also reveal differences between genetic inactivation of PHDs, responses to hypoxia and responses to a pharmacological inhibitor, demonstrating the need for caution in predicting the effects of therapeutic modulation of the HIF hydroxylase system on different physiological responses.

**Key points**

- Arterial hypoxaemia leads to a rapid increase in ventilation. If the hypoxaemia is sustained, a further increase in ventilation develops over hours to days in a process termed ventilatory acclimatisation.
- Studies in transgenic mice implicate the hypoxia-inducible factor (HIF) pathway in the latter process.
- The aim of this study was to investigate the role of HIF prolyl hydroxylase (PHD) enzymes in ventilatory acclimatisation.
- We find that *PHD2*^+/−^, but not *PHD1^−/−^* or *PHD3^−/−^*, mice mimic chronic hypoxia in exhibiting exaggerated ventilatory responses to acute hypoxia. This was associated with carotid body overgrowth. However, use of a PHD inhibitor (PHI) induced both hypoxic ventilatory sensitivity and carotid body proliferation only marginally despite strongly inducing erythropoiesis.
- Taken together, these findings implicate HIF/PHD2 in ventilatory control and carotid body biology but highlight the difficulty of translation from genetic models to pharmacological intervention.

## Introduction

Acute exposure to hypoxia elicits a rapid increase in ventilation that occurs within seconds, followed by a further progressive increase in ventilation developing over a period of hours to days. It has been established that this latter effect, termed acute ventilatory acclimatisation, is in part due to enhanced ventilatory sensitivity to hypoxia itself (Prabhakar & Jacono, 2005; Robbins, 2007). Although this phenomenon was first observed by J. S. Haldane and colleagues in their classical studies of ventilatory responses to altitude conducted over 100 years ago (Douglas *et al.* 1913), the underlying mechanisms remain poorly understood.

It is now recognised that many cellular and systemic responses to hypoxia are mediated by the HIF hydroxylase system, in which the oxygen-sensitive signal is generated by impaired catalysis of a set of 2-oxoglutarate-dependent dioxygenases (Kaelin & Ratcliffe, 2008; Prabhakar & Semenza, 2012). These enzymes catalyse the oxygen-dependent post-translational hydroxylation of HIF-α subunits to regulate both the stability and activity of the transcriptional complex. In mammalian systems HIF-α presents as three isoforms of which two, HIF-1α and HIF-2α, are the most studied. Despite binding a similar DNA consensus, HIF-1α and HIF-2α have only partially overlapping transcriptional targets and have distinct biological actions in many settings (Hu *et al.* 2003; Schödel *et al.* 2011). The stability of both HIF-1α and HIF-2α is regulated by prolyl hydroxylation which promotes degradation by the von Hippel–Lindau (VHL) ubiquitin E3 ligase and degradation by the ubiquitin–proteasome pathway (Kaelin & Ratcliffe, 2008; Prabhakar & Semenza, 2012). This reaction is catalysed by three related prolyl hydroxylase domain (PHD) enzymes that exhibit partial selectivity for different HIF-α isoforms in cells. PHD2 is the most abundant enzyme in the majority of tissues and is also the most important in setting normoxic levels of HIF-1α (Berra *et al.* 2003; Appelhoff *et al.* 2004). In contrast, PHD1 and PHD3 are usually expressed at lower levels and make a greater contribution to the regulation of HIF-2α (Appelhoff *et al.* 2004; Aragones *et al.* 2008; Schneider *et al.* 2010). Inhibition of the PHD enzymes by small molecules that compete with 2-oxoglutarate for binding at the PHD-active site leads to strong activation of the HIF transcriptional response even in normoxic cells. This has generated widespread interest in the pharmaceutical development of such inhibitors for anaemia, ischaemic diseases and other conditions where augmentation of hypoxic signalling may be beneficial (Hsieh *et al.* 2007; Fraisl *et al.* 2009; Bernhardt *et al.* 2010; Yan *et al.* 2010; Rose *et al.* 2011).

Physiological analyses of mice with homozygous inactivation of HIF-1α or HIF-2α are confounded by embryonic lethal or severe neonatal/developmental phenotypes (Iyer *et al.* 1998; Tian *et al.* 1998; Kotch *et al.* 1999; Peng *et al.* 2000; Scortegagna *et al.* 2003). Analyses of the effects of HIF on ventilatory control have therefore been conducted in mice with heterozygous deletion of HIF-1α or HIF-2α, because they are viable and manifest few abnormalities in the basal state. Interestingly, studies of these animals have revealed different ventilatory phenotypes. *HIF-1α*^+/−^ animals showed unaltered responses to acute hypoxia (Kline *et al.* 2002; Peng *et al.* 2006). Moreover, the secretory response to acute hypoxia is unaltered in carotid body slices obtained from *HIF-1α*^+/−^ animals (Ortega-Saenz *et al.* 2007). Nevertheless, *HIF-1α*^+/−^ animals manifest severe reduction in the enhancement of ventilatory sensitivity that follows exposure to either chronic sustained or chronic intermittent hypoxia (Kline *et al.* 2002; Peng *et al.* 2006). In contrast, *HIF-2α*^+/−^ mice manifest an increase in their basal ventilatory sensitivity to acute hypoxia and irregular breathing patterns (Peng *et al.* 2011).

Although these studies implicate the HIF system in the regulation of ventilatory sensitivity to hypoxia, they leave open important questions as to whether and how the activity of the ‘oxygen-sensing’ PHD enzymes that regulate HIF (and potentially the activity of other substrates) affects either acute responses to hypoxia or the modulation of ventilatory sensitivity that occurs in response to prior hypoxic exposure. To address this, we studied ventilatory responses to hypoxia in mice bearing inactivating alleles of each of the PHD enzymes and in mice exposed to a small molecule prolyl hydroxylase inhibitor (PHI), 2-(1-chloro-4-hydroxyisoquinoline-3-carboxamido) acetic acid (IOX3). We then compared responses to those induced by prior exposure to sustained hypoxia. Animals with deficiency in PHD2, but not PHD1 or PHD3, manifest striking increases in ventilatory sensitivity and carotid body hyperplasia that are similar to those observed after exposure to 7 days of sustained hypoxia. In contrast, PHI had no immediate effects on ventilation and caused only slight increases in ventilatory sensitivity and carotid body hyperplasia after 7 days exposure, despite efficient stimulation of erythropoiesis. These findings together indicate that some, but not all, of the effects of hypoxia on ventilatory control are mediated by PHD enzymes and that PHD2 is the most important enzyme mediating these effects.

## Methods

### Animals

Male mice, approximately 3 months old, from the same litter were used for all comparisons. Chronic hypoxia and IOX3-treated wild-type mice, as well as *PHD2*^+/−^ mice (Mazzone *et al.* 2009), were on a pure C57BL/6 genetic background; *PHD1^−/−^* (Aragones *et al.* 2008) and *PHD3^−/−^* (Bishop *et al.* 2008) mice were on a mixed Swiss/129SvEv genetic background. All animal procedures were compliant with the UK Home Office Animals (Scientific Procedures) Act 1986 and Local Ethical Review Procedures (University of Oxford Medical Sciences Division Ethical Review Committee).

### Pre-exposure of animals to hypoxia and bromodeoxyuridine labelling

Animals were exposed to 10% oxygen for 7 days in an adapted isolator chamber designed to maintain 10% oxygen levels whilst tightly controlling temperature, humidity and carbon dioxide levels. Control animals were maintained in room air over the same period. Mice were treated with bromodeoxyuridine (BrdU; Sigma) as described (Pardal *et al.* 2007): 50 mg kg^−1^ intraperitoneal injection on day 1, followed by supplementation of the drinking water with 1 mg ml^−1^ for the duration of the experiment.

### Ventilatory measurements

Tidal volume and respiratory rate were measured in awake animals using individual whole body plethysmographs (600 ml volume; Model PLY4211, Buxco, Wilmington, NC, USA). Minute ventilation was calculated from tidal volume and respiratory rate. Premixed gas was delivered to each chamber at 2 l min^−1^. The hypoxic stimulus consisted of 10% oxygen, balance nitrogen or 10% oxygen, 3% carbon dioxide, balance nitrogen. Before exposure to hypoxia, mice were allowed to breathe air within the chamber for at least 30 min; ventilation was then measured over 5 min of breathing air for baseline measurements. For quantification, the hypoxic ventilatory response (HVR) was defined as the difference between minute ventilation (or respiratory rate or tidal volume; see Supplemental Tables 1–4, available online only) during the 1 min prior to the onset of hypoxia and the first 1 min of stable hypoxia (i.e. excluding the first 30 s of hypoxia).

### Pulse oximetry

Arterial oxygen saturation in conscious, unrestrained mice was measured using a mouse pulse oximeter (MouseOx, Starr Life Sciences, Harvard Apparatus: Edenbridge, UK).

### Blood measurements

Blood samples were obtained from the tail vein of mice using heparinised capillary tubes. Haematocrits were measured using a haematocrit centrifuge (Model C-MH30, Unico, Dayton, NJ, USA). Equal volumes of 0.1% Brilliant Cresyl Blue (Sigma) and blood were mixed and left at room temperature for 20 min before smearing onto slides for reticulocyte counts.

### Histological and stereological analyses

Carotid bodies were harvested from mice under terminal anaesthesia (isoflurane followed by exsanguination). These were fixed in 4% paraformaldehyde overnight then transferred into 70% ethanol, processed, wax-embedded and sectioned to 4 μm thickness. To assess carotid body morphometry, sections were blocked for 1 h at room temperature with protein serum then incubated overnight at 4°C with a rabbit anti-tyrosine hydroxylase (TH) polyclonal antibody (Novus Biologicals, Cambridge, UK). Sections were washed four times in PBS–Triton then incubated with goat anti-rabbit secondary antibody (Envision+, Dako, Cambridge, UK). Stereological estimation of TH-positive cells, carotid body volume, TH-positive cell density and TH-positive cell volume was performed on alternate sections throughout the organ using ImagePro Plus software. To estimate BrdU-positive cell counts, sections were immunostained with an antibody against BrdU according to the manufacturer's instructions (Becton Dickinson, Oxford, UK). Adjacent sections were immunostained with TH as above to delineate the carotid body. Stereological estimation of BrdU-positive cells was performed using ImagePro Plus software.

### IOX3 treatment of animals

Animals were injected intraperitoneally with a bi-cyclic isoquinolinyl prolyl hydroxylase inhibitor 2-(1-chloro-4- hydroxyisoquinoline-3-carboxamido) acetic acid (IOX3) (Tian *et al.* 2011): 30 mg kg^−1^ in Hanks’ buffered saline solution (HBSS) with 5% DMSO, pH 7, twice daily for 7 days (∼100 μl per injection or ∼1.4 ml in total over the 7 days); control animals were injected with vehicle only. Mice were simultaneously treated with BrdU to assay cell proliferation, as described above. Both control and IOX3-treated mice appeared healthy and no ill effects were observed either during or following treatment.

### Statistical analysis

Data are presented as mean ± SEM. For *PHD2*^+/−^, *PHD1^−/−^* and *PHD3^−/−^* mice, differences in HVR compared with respective littermate control groups were made using unpaired Student's *t* tests. To assess the effects of chronic hypoxia, HVR was measured on day 1 (i.e. before the onset of chronic hypoxia), and again on day 7 of the exposure. The change in HVR in the chronic hypoxia group was compared with the corresponding change in the control (air-breathing) group using an unpaired Student's *t* test. The same approach was used to assess the change in HVR after 7 days of IOX3, compared with the corresponding change in the control (vehicle) group. *P* values <0.05 were considered statistically significant.

## Results

The aim of this study was to determine the extent to which the effects of ventilatory acclimatisation to hypoxia could be mimicked by genetic and pharmacological manipulation of the oxygen-sensing HIF prolyl hydroxylase domain (PHD) enzymes.

### Ventilatory acclimatisation to hypoxia in wild-type mice

**Figure 1:**
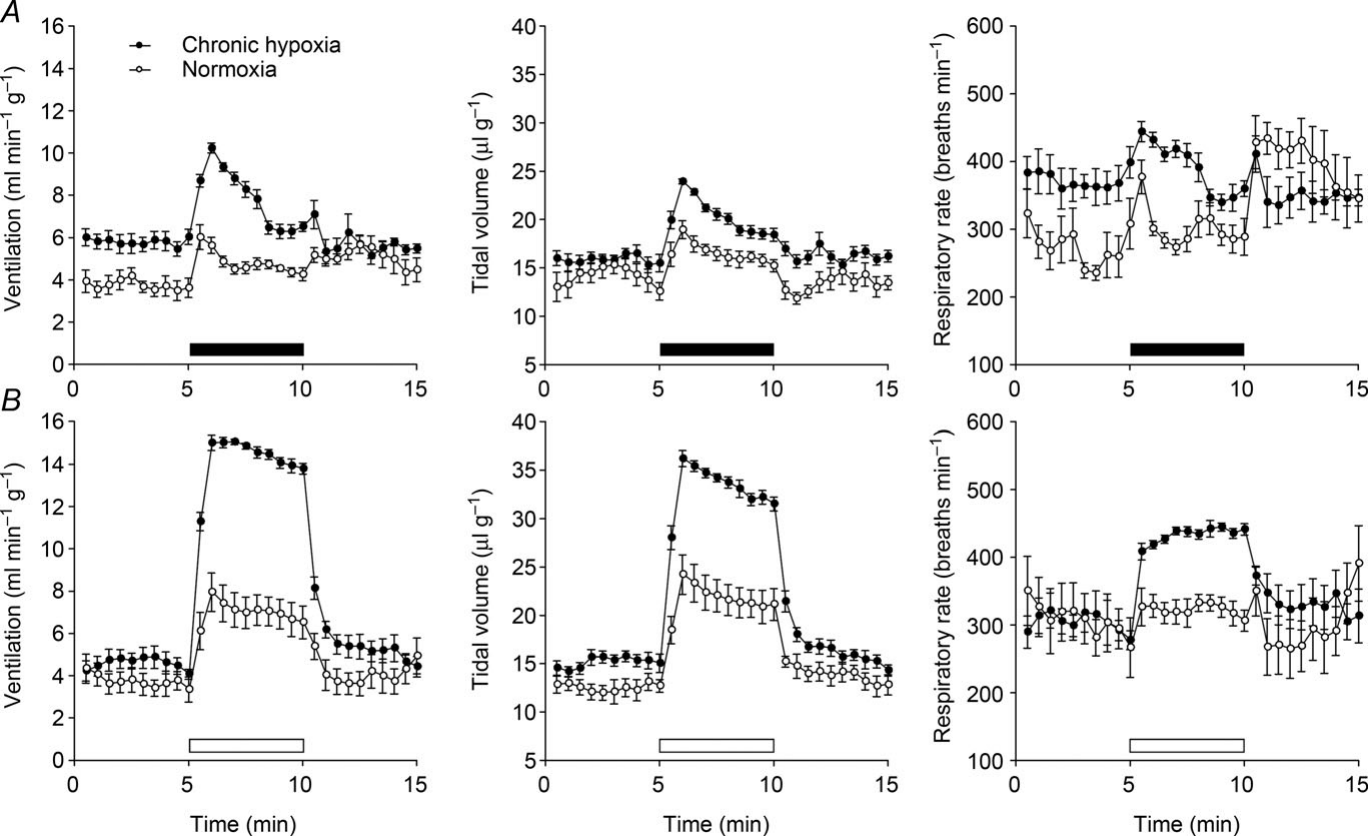
Effect of pre-exposure to chronic hypoxia on ventilatory responses to acute hypoxia Minute ventilation (left), tidal volume (middle) and respiratory rate (right) before, during and after an acute hypoxic stimulus, in mice pre-exposed to continuous hypoxia (10% oxygen) for 7 days or maintained in normoxia. Acute exposure to 10% oxygen was made without (*A*) and with (*B*) the addition of 3% carbon dioxide (filled and open bars, respectively). Mean ± SEM; *n*= 8 for each group.

To provide a framework for the study we first tested the effects of chronic hypoxia on ventilatory responses to acute hypoxic stimuli. Mice were accordingly subjected to 10% oxygen in a normobaric chamber for 7 days and then placed, unrestrained, in a whole animal plethysmograph breathing normal air for 30 min, before being subjected to an acute hypoxic stimulus for 5 min. Ventilation was analysed before, during and after this stimulus. Responses were compared with littermate control mice maintained in normoxia. In control animals, acute exposure to hypoxia (10% oxygen) elicited an immediate but poorly sustained increase in ventilation, termed the hypoxic ventilatory response (HVR; Fig. 1*A* and Supplemental Table 1). In contrast, mice pre-exposed to chronic hypoxia exhibited an enhanced HVR (Fig. 1*A* and Supplemental Table 1), as well as a variable increase in basal ventilation.

In humans, attenuation of HVR due to respiratory alkalosis can partly be prevented by clamping of the end-tidal carbon dioxide level (which is otherwise reduced by the increase in ventilation; Howard & Robbins, 1995), but this procedure is not possible in mice. Instead, we sought to offset the effect of increased ventilation on carbon dioxide levels by adding a small concentration of carbon dioxide to the hypoxic atmosphere used for acute stimulation. We added 3% carbon dioxide to the hypoxic gas mixture based on published data which shows that adding this to inspired gas prevents a major rise in arterial pH or fall in 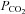 during exposure of mice to 7% oxygen (Ishiguro *et al.* 2006). Exposure of mice to 10% oxygen with 3% carbon dioxide produced a larger and better-sustained increase in ventilation in control mice (Fig. 1*B* and Supplemental Table 1). Furthermore, the enhanced response following 7 days pre-exposure to hypoxia was very clearly apparent (Fig. 1*B* and Supplemental Table 1) with striking increases in both the tidal volume and respiratory rate. Overall, HVR was more than 2-fold greater in the group of animals exposed to 7 days of hypoxia (10.8 ± 0.3 ml min^−1^ g^−1^) than in the control group (4.1 ± 0.7 ml min^−1^ g^−1^) (Fig. 1*B* and Supplemental Table 1). These results confirm that mice, like other species, manifest an exaggerated respiratory response to acute hypoxia following a sustained period of chronic hypoxia.

### Enhanced hypoxic ventilatory responses in *PHD2*^+/−^ mice

We next sought to test whether exaggerated respiratory responses were observed in mice bearing inactivating alleles of the PHD oxygen-sensing enzymes 1, 2 and 3. PHD2 is the most abundant PHD in most tissues and is also the most important in the regulation of the HIF-1α isoform (Berra *et al.* 2003; Appelhoff *et al.* 2004). *PHD2^−/−^* mice are embryonic lethal (Takeda *et al.* 2006), but *PHD2*^+/−^ are viable, fertile and phenotypically near normal in the basal state, in spite of reduced PHD2 levels (Mazzone *et al.* 2009) and were therefore used in these studies.

**Figure 2:**
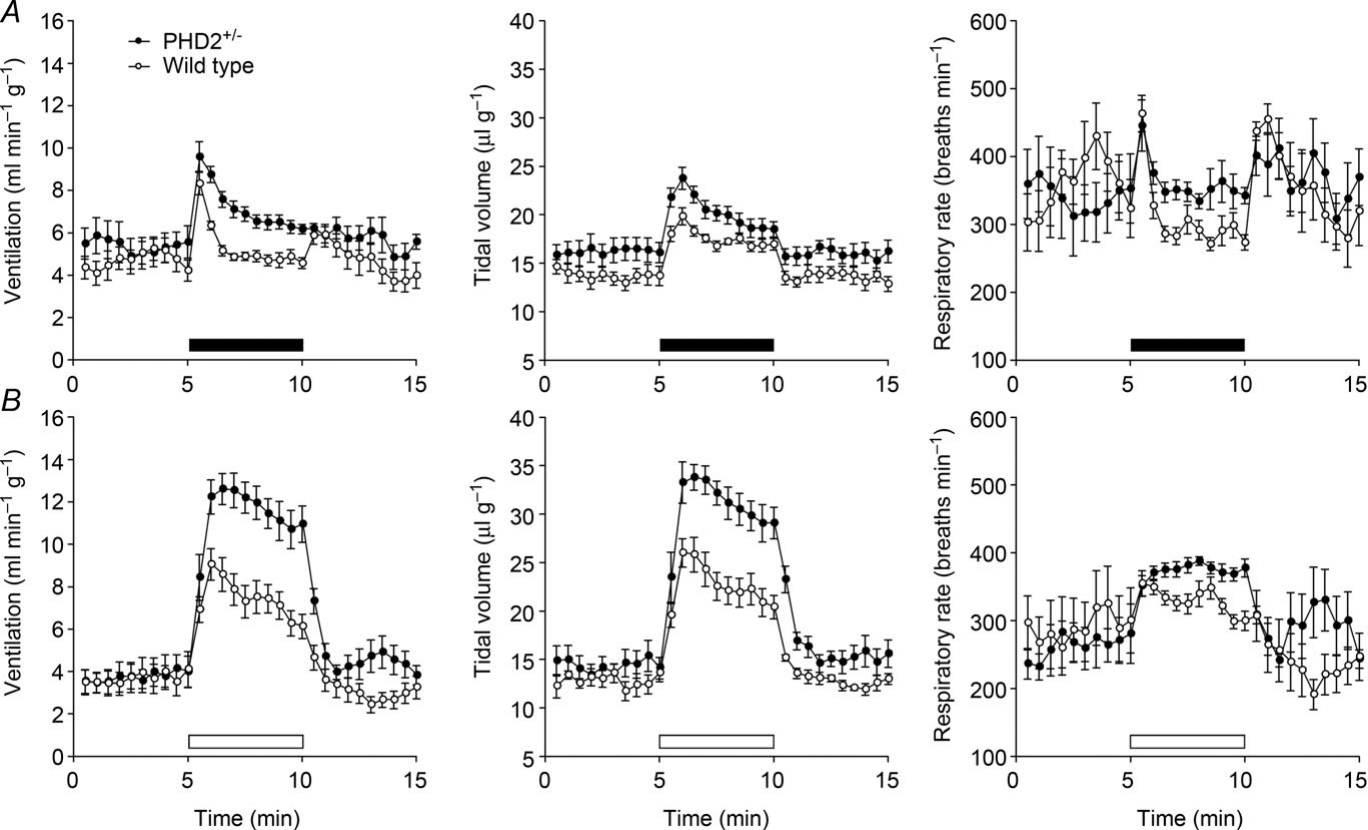
Ventilatory responses to acute hypoxia in *PHD2*^+/−^ and littermate control wild-type mice Minute ventilation (left), tidal volume (middle) and respiratory rate (right) after acute exposure to 10% oxygen without (*A*) and with (*B*) the addition of 3% carbon dioxide (filled and open bars, respectively). Mean ± SEM; *n*= 7 for each group.

Ventilatory responses in *PHD2*^+/−^ mice were tested using both gas mixtures described above: 10% oxygen (Fig. 2*A*) and 10% oxygen with 3% carbon dioxide (Fig. 2*B*). A large increase in response to the latter stimulus was observed in *PHD2*^+/−^ mice compared with wild-type littermate controls (Fig. 2*B* and Supplemental Table 2). This was similar in character to that observed after chronic hypoxia pre-treatment and involved mainly an increase in tidal volume (Fig. 2*B*, middle; Supplemental Table 2). Unlike after chronic hypoxia treatment, however, baseline ventilation was not enhanced in *PHD2*^+/−^ mice (Fig. 2).

**Figure 3:**
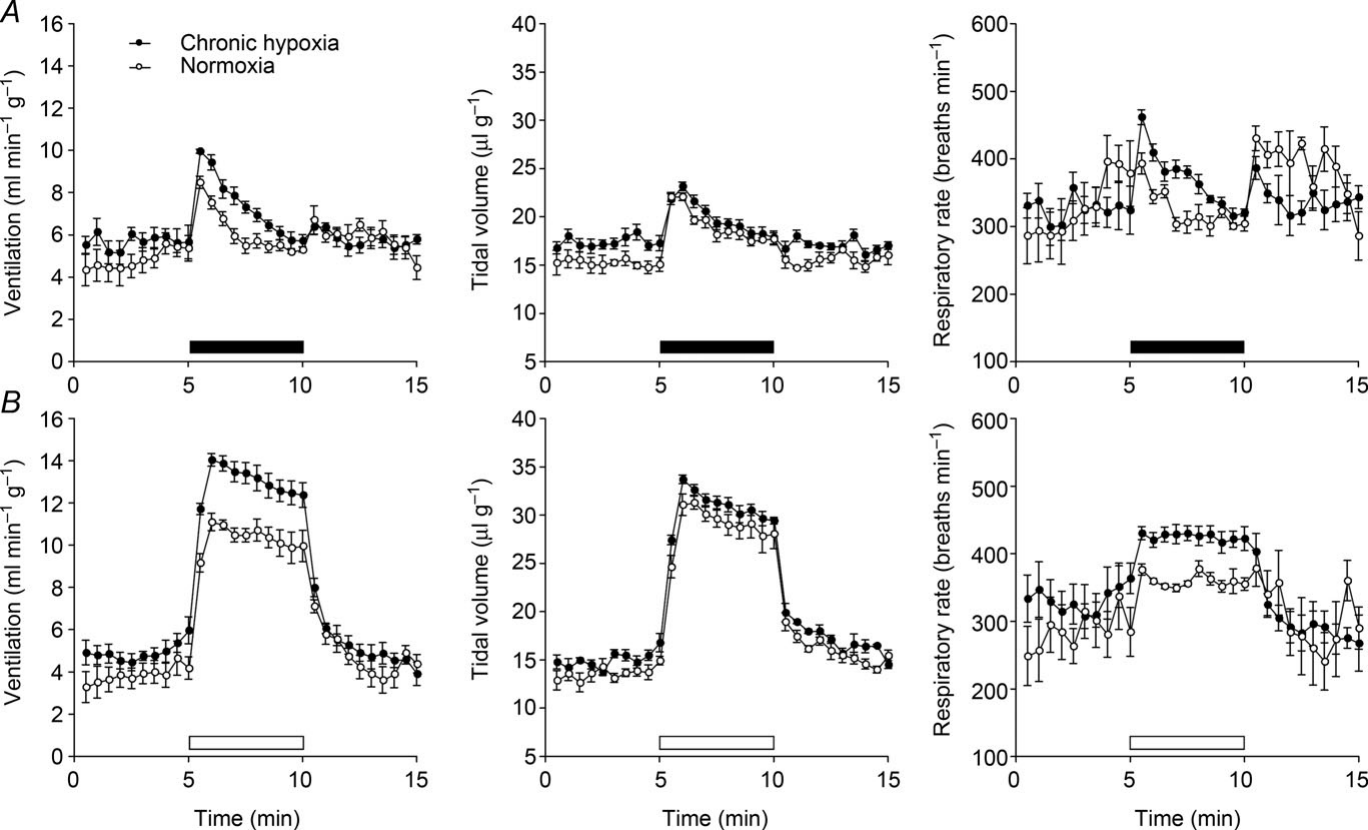
Effect of pre-exposure to chronic hypoxia on ventilatory responses to acute hypoxia in *PHD2*^+/−^ mice Minute ventilation (left), tidal volume (middle) and respiratory rate (right) before, during and after an acute hypoxic stimulus, in *PHD2*^+/−^ mice pre-exposed to continuous hypoxia (10% oxygen) for 7 days or maintained in normoxia. Acute exposure to 10% oxygen was made without (*A*) and with (*B*) the addition of 3% carbon dioxide (filled and open bars, respectively). Mean ± SEM; *n*= 5 for each group.

To assess whether the effects of PHD2 inactivation were additive to those of chronic hypoxia, *PHD2*^+/−^ mice were exposed to 7 days of 10% oxygen (or normoxia in littermate controls) using the protocol described above for wild-type mice. There was a small, non-significant increase in HVR following chronic hypoxia exposure of *PHD2*^+/−^ mice (Fig. 3 and Supplemental Table 3). This was observed with both acute hypoxic exposures: 10% oxygen (Fig. 3*A*) and 10% oxygen with 3% carbon dioxide (Fig. 3*B*). Comparison of the absolute HVR after 7 days pre-exposure to hypoxia in these *PHD2*^+/−^ mice with those of wild-type mice after similar 7 day pre-exposure to hypoxia (Fig. 1 and Supplemental Table 1) revealed similar values, indicating that augmentation of HVR by genetic inactivation of PHD2 and hypoxic pre-exposure were non-additive.

Interestingly, *PHD2*^+/−^ mice manifest only a very slight elevation of haematocrit (Table 1) despite markedly enhanced ventilatory responses. This is in contrast with chronic hypoxia exposure, which resulted in striking effects on erythropoiesis (increased haematocrit and reticulocyte counts, Table 1).

**Table 1:** Haematological parameters

	Wild-type		*PHD2*^+/−^					
						
Treatment	Normoxia	Hypoxia	Normoxia	Hypoxia	Wild-type	*PHD2*^+/−^	Vehicle	IOX3
Haematocrit (%)	49 ± 1	68 ± 1***	50 ± 1	63 ± 1**	49 ± 1	53 ± 1*	48 ± 1	59 ± 1***
Reticulocyte count (% of red blood cells)	1.3 ± 0.1	7.3 ± 1.0***	1.7 ± 0.5	8.3 ± 0.9**	1.7 ± 0.2	1.9 ± 0.2	1.9 ± 0.2	5.8 ± 0.4***

Haematocrit and reticulocyte counts (%) in: (i) wild-type mice exposed to hypoxia (10% oxygen) or normoxia for 7 days; (ii) *PHD2*^+/−^ mice exposed to hypoxia (10% oxygen) or normoxia for 7 days; (iii) wild-type and *PHD2*^+/−^ mice; (iv) wild-type mice treated with IOX3 or vehicle for 7 days. Mean ± SEM; *n*= 8 (wild-type: normoxia and chronic hypoxia); *n*= 5 (*PHD2^+/^*^−^: normoxia and chronic hypoxia); *n*= 8 (wild-type; *PHD2*^+/−^); and *n*= 11 (vehicle; IOX3) mice in each group; ***P < 0.0001; **P < 0.01; *P < 0.05.

**Figure 4:**
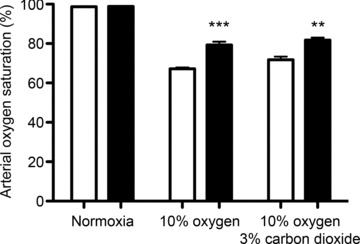
Arterial oxygen saturation in acute hypoxia Conscious, unrestrained *PHD2*^+/−^ animals (filled bars) and littermate control wild-type animals (open bars); animals in normoxia, 10% oxygen or 10% oxygen with 3% carbon dioxide. Mean ± SEM; *n*= 5 mice for each group; ***P < 0.0001; **P < 0.01.

Arterial oxygen saturations were measured during hypoxia in both groups of mice to exclude the possibility that increased ventilatory responses in *PHD2*^+/−^ mice were the result of greater hypoxaemia arising from an unknown effect of PHD2 on pulmonary gas exchange. In keeping with enhanced ventilatory responses, however, *PHD2*^+/−^ mice maintained higher levels of blood oxygen during different types of acute hypoxia stimulation (Fig. 4). These observations confirm that enhanced responses reflect a genuine increase in ventilatory sensitivity, not a greater level of stimulation.

### Carotid body overgrowth in *PHD2*^+/−^ mice

**Figure 5:**
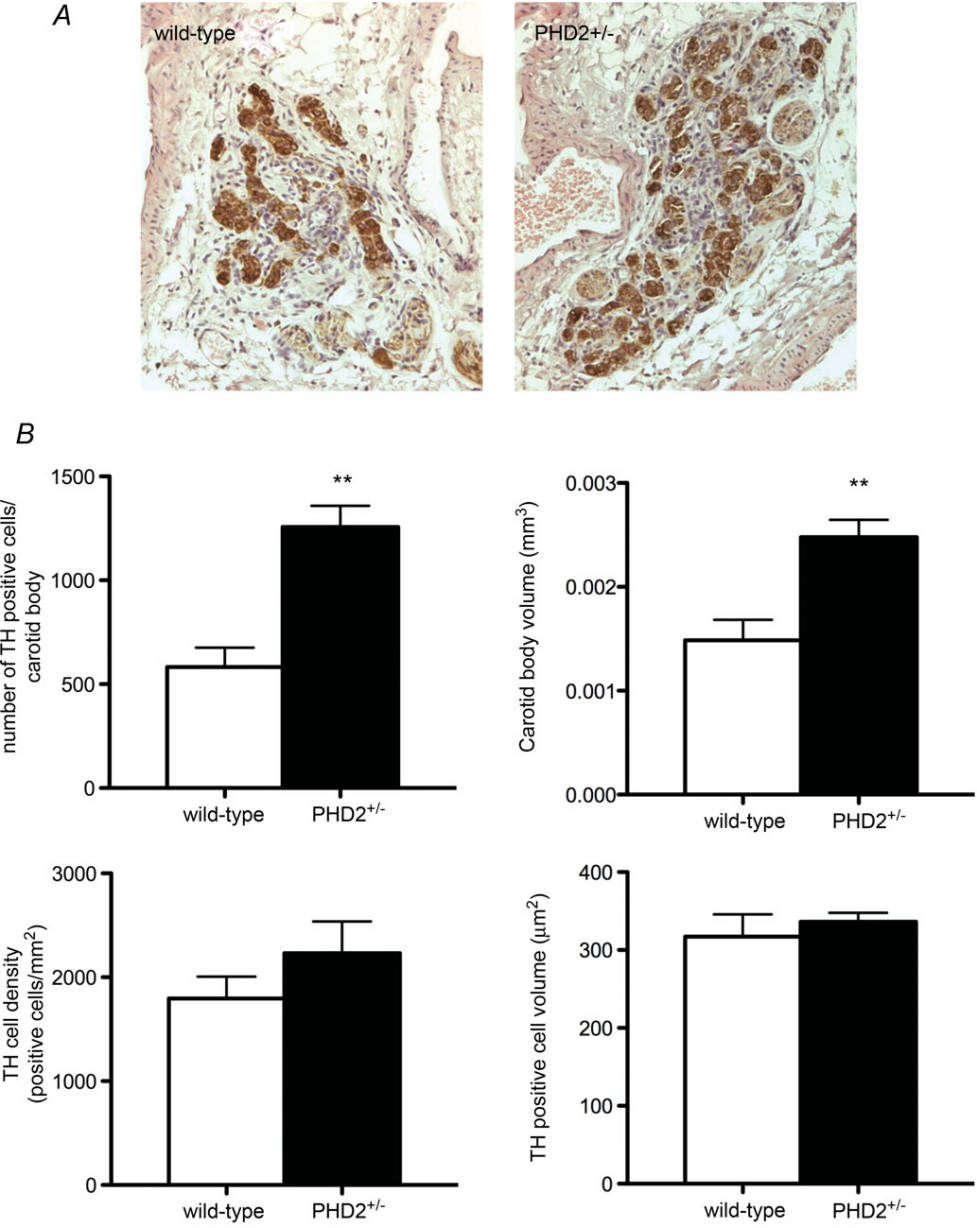
Histological analysis of carotid bodies *A*, representative sections of wild-type and *PHD2*^+/−^ carotid bodies (CB) immunostained with tyrosine hydroxylase (TH) (brown). *B*, morphometric analysis of TH-positive cells, carotid body volume, TH-positive cell density and TH-positive cell volume in the CB. Mean ± SEM; *n*= 5 mice for each group; **P < 0.01.

Since increased ventilatory sensitivity after hypoxic exposure has been associated with growth of the carotid body in a range of species (Kay & Laidler, 1977; McGregor *et al.* 1984), we next analysed both morphologically and morphometrically carotid bodies from *PHD2*^+/−^ mice (Fig. 5). Carotid body sections were immunostained with anti-tyrosine hydroxylase (TH) antibodies, as a marker of type I cells. Morphometric analysis demonstrated a clear increase in type I cells in *PHD2*^+/−^ mice, with a doubling in TH-positive cell number and carotid body volume, but no change in TH-positive cell density or TH-positive cell size (Fig. 5*B*). Hyperplastic type I cells appeared essentially normal, with well-defined glomeruli and no evidence of dysplasia (Fig. 5*A*). Thus, *PHD2*^+/−^ mice show both marked carotid body hyperplasia and increased ventilatory sensitivity.

### Hypoxic ventilatory responses in *PHD3^−/−^* and *PHD1^−/−^* mice

We have reported that carotid bodies from *PHD3^−/−^* mice have an increased number of TH-positive cells (Bishop *et al.* 2008). Unlike the *PHD2*^+/−^ carotid bodies, the overgrowth in *PHD3^−/−^* carotid bodies occurred without a corresponding increase in carotid body volume. Furthermore, this qualitatively abnormal overgrowth was observed in other sympathoadrenal tissues, such as the adrenal medulla and supracervical ganglion, which were hypofunctional in spite of the overgrowth (Bishop *et al.* 2008).

**Figure 6:**
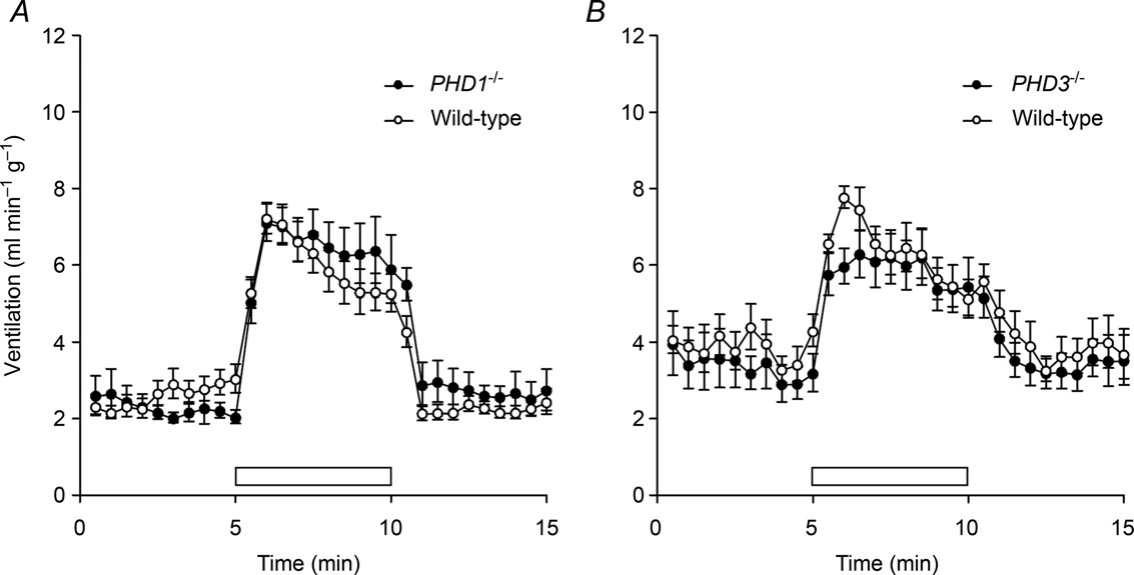
Ventilatory responses of *PHD1*^−/−^ and *PHD3*^−/−^ mice Changes in minute ventilation in response to 10% oxygen with 3% carbon dioxide in *PHD1^−/−^* (*A*) and *PHD3^−/−^* (*B*) and littermate control wild-type mice. Acute exposure to hypoxia: 10% oxygen with 3% carbon dioxide (open bars). Mean ± SEM; *n*= 7 for each group (*A*); *n*= 5 for each group (*B*).

In view of these findings and the ventilatory responses observed in the *PHD2*^+/−^ mice, we proceeded to test ventilatory responses in both *PHD3^−/−^* and *PHD1^−/−^* mice. Based on the clear ventilatory responses obtained using 10% oxygen with 3% carbon dioxide above, *PHD1^−/−^* and *PHD3^−/−^* mice were tested using the same stimulus. In contrast to *PHD2*^+/−^ mice, the hypoxic ventilatory response to this gas mixture was unaltered in both *PHD1^−/−^* and *PHD3^−/−^* mice (Fig. 6*A* and *B*; Supplemental Table 2). These results indicate that partial deficiency of PHD2 – the principal enzyme acting on HIF-1α– had the clearest effects in mimicking chronic hypoxia by inducing ventilatory sensitivity. This is despite the finding that PHD1 and PHD3 are expressed in the carotid body and neuroendocrine tissues and can influence the morphology of these tissues (Bishop *et al.* 2008; Pan *et al.* 2012).

### Pharmacological inhibition of PHD enzymes produces modest increases in hypoxic ventilatory sensitivity and carotid body proliferation

Since PHD inhibitors (PHI) are in clinical trials for anaemia (for example: http://www.clinicaltrials.gov/ct2/show/NCT00456053), we considered whether they might have additional effects (desirable or otherwise) on ventilatory sensitivity.

**Figure 7:**
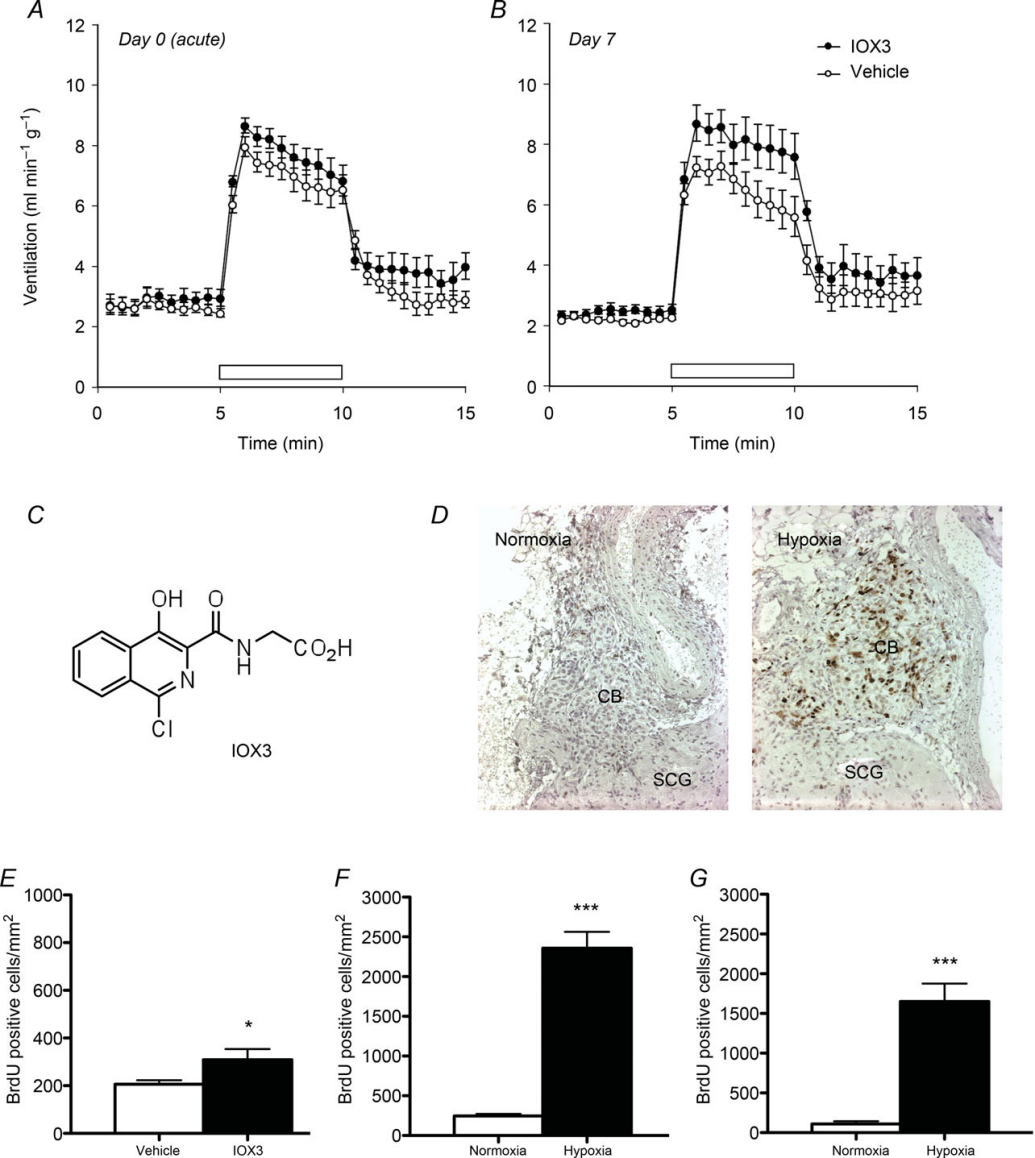
Comparison of effects of IOX3 treatment *versus* continuous hypoxia Mice were treated with IOX3 (30 mg kg^−1^ twice daily) or vehicle (HBSS with 5% DMSO) for 7 days. Minute ventilation in response to 10% oxygen with 3% carbon dioxide (open bars) in IOX3-treated or vehicle-treated animals immediately following (*A*) or 7 days after (*B*) the first injection. *C*, chemical structure of IOX3. *E*, carotid body BrdU incorporation after 7 days of IOX3 treatment; mean ± SEM; *n*= 11 for each group; *P < 0.05. *D*, *F* and *G*, carotid body proliferation in mice exposed to continuous hypoxia (10% oxygen) or normoxia for 7 days. Morphometric analysis of BrdU staining in CBs from wild-type (*F*) and *PHD2*^+/−^ (*G*) mice; mean ± SEM; *n*= 8 (*F*) or 5 mice (*G*) for each group; ***P < 0.0001. *D*, representative images of BrdU staining (brown) in wild-type mice; CB: carotid body; SCG: supracervical ganglion.

We therefore treated mice with 2-(1-chloro-4- hydroxyisoquinoline-3-carboxamido) acetic acid (IOX3, Fig. 7*C*), a potent inhibitor of the human PHD enzymes with selectivity over at least some other 2-oxoglutarate-dependent dioxygenases including factor inhibiting HIF (FIH) (Tian *et al.* 2011). This compound has been assigned as FG-2216 (Yan *et al.* 2010), a compound that has been progressed to Phase II clinical trials in man for the treatment of anaemia (FibroGen, Inc., Treatment method for anaemia. US20060276477; 2006). IOX3 is identical to ICA, a compound which has been shown to induce HIF strongly in rodent kidney following administration at 25–50 mg kg^−1^ (Schley *et al.* 2012; Wang *et al.* 2012).

In pilot experiments, we confirmed that a dose of 30 mg kg^−1^ was sufficient to induce HIF in the liver near maximally (Supplemental Fig. 1). Although 60 mg kg^−1^ induced marginally higher levels of HIF, prolonged administration of this dose was poorly tolerated. In contrast, administration of 30 mg kg^−1^ twice daily was well tolerated and induced a robust increase in blood reticulocytes in mice, confirming the anticipated stimulatory action on erythropoiesis that has been demonstrated in rodents and primates using an equivalent daily dose (Hsieh *et al.* 2007).

To test for effects on ventilatory sensitivity, we treated animals for a total of 7 days to permit direct comparison with the effects of exposure to hypoxia. We measured responses to the standard 5 min acute exposure to 10% hypoxia with 3% carbon dioxide before and immediately after the first dose and at intervals of 1, 2, 3, 5 and 7 days after the start of treatment (Fig. 7 and Supplemental Fig. 2). To permit further comparisons with responses to hypoxia (Pardal *et al.* 2007), we also measured carotid body cellular proliferation by BrdU incorporation and compared results at 7 days with those obtained in animals that had been exposed to hypoxia (10% oxygen) for 7 days.

As expected, treatment with PHI over the 7 day protocol induced a striking reticulocytosis and increase in haematocrit, which was broadly similar to that induced by the 7 day exposure to hypoxia (Table 1). PHD inhibition had no immediate effect on basal respiration or sensitivity to the acute hypoxic stimulus (10% oxygen in the presence of 3% carbon dioxide) (Fig. 7*A* and Supplemental Table 4). After 7 days, however, PHD inhibition did modestly increase responsiveness to the acute hypoxic stimulus (Fig. 7*B* and Supplemental Table 4) and also slightly induced carotid body cell proliferation (Fig. 7*E*). Despite similar effects on erythropoiesis, however, the effects of PHD inhibition on ventilatory sensitivity and carotid body cell proliferation were substantially smaller than those observed after exposure to chronic hypoxia (Figs 1*B* and 7).

## Discussion

This study demonstrates that heterozygous inactivation of the ‘oxygen-sensing’ enzyme PHD2 is associated with markedly enhanced ventilatory sensitivity to hypoxia. *PHD2*^+/−^ animals manifest large increases in ventilatory sensitivity to hypoxia despite only modest apparent dysregulation of oxygen homeostasis in the basal state (for instance a very slight increase in haematocrit). When compared to littermate controls, *PHD2*^+/−^ animals responded to acute hypoxia in an exaggerated manner that was quantitatively similar to that observed after pre-exposure to a 7 day period of continuous hypoxia. In contrast, animals with homozygous inactivation of other PHD isoforms, *PHD1^−/−^* and *PHD3^−/−^*, behaved normally, despite the abnormalities of carotid body morphology that we have previously reported in *PHD3^−/−^* animals (Bishop *et al.* 2008).

Enhanced ventilatory sensitivity to hypoxia in *PHD2*^+/−^ mice was associated with alterations in carotid body morphology that were qualitatively distinct from those observed in *PHD3^−/−^* mice. Morphological and morphometry analyses using anti-tyrosine hydroxylase antibodies revealed increased numbers of morphologically normal type I cells in *PHD2*^+/−^ mice, resulting in an approximately 60–70% increase in carotid body volume; changes which are similar to those that have been reported in animals exposed to chronic hypoxia (Laidler & Kay, 1975; Pardal *et al.* 2007). Despite the clear involvement of PHD3 in sympathoadrenal development and function (Bishop *et al.* 2008), the current work suggests that PHD2 is the most important PHD isoform regulating the classically observed adaptive changes in ventilatory control in response to sustained hypoxia.

Whereas PHD2 is the most abundant of the PHD enzymes and makes the most important contribution to the setting of normoxic (basal) levels of HIF-1α, several studies have indicated that PHD1 and PHD3 make a somewhat greater contribution to the regulation of HIF-2α (Appelhoff *et al.* 2004; Aragones *et al.* 2008; Schneider *et al.* 2010). Studies of ventilatory control in mice that are heterozygous for different HIF-α isoforms reveal contrasting phenotypes in *HIF-1α* heterozygous *versus HIF-2α* heterozygous animals. Isolated carotid bodies from heterozygous *HIF-1α* mice manifest reduced sensitivity to hypoxia and *HIF-1α*^+/−^ animals also exhibit reduced ventilatory responses to some, but not all, forms of hypoxic stimulation (Kline *et al.* 2002; Peng *et al.* 2006). In contrast, *HIF-2α*^+/−^ mice manifest a quite different phenotype involving exaggerated responses to hypoxia and irregular resting respiration (Peng *et al.* 2011). These findings suggest that predicting the overall output of modulating the HIF/PHD system is likely to be complex. In keeping with this, Chuvash polycythaemia is a recessive condition caused by bi-allelic inheritance of a hypomorphic VHL allele that impairs degradation of both HIF-α subunits, leads to basal arterial hypocapnia, and markedly increases ventilatory responses to hypoxia (Smith *et al.* 2006). Individuals with erythrocytosis secondary to gain-of-function mutations in HIF-2α, however, manifest basal hypocapnia with little or no change in acute ventilatory sensitivity to hypoxia (Formenti *et al.* 2011).

To test responses to pharmacological inhibition of the PHD enzymes directly, a 2-oxoglutarate competitor suitable for use in animals (IOX3) (Tian *et al.* 2011) was administered to mice in doses that engendered a rapid erythrocytosis. These were compared with the adaptive response to continuous hypoxia over a similar period of time, by measuring both changes in ventilatory sensitivity to acute hypoxia and effects on cellular proliferation within the carotid body. Several interesting findings were noted. First, when measured immediately after administration of IOX3, no effect could be discerned on either basal ventilation or the response to acute hypoxia, consistent with the absence of effect of the non-specific 2-oxoglutarate-dependent dioxygenase inhibitor dimethyloxalylglycine on the response of carotid body slices to acute hypoxia (Ortega-Saenz *et al.* 2007). Second, after 7 days, a significant increase in both ventilatory sensitivity and cellular proliferation within the carotid body was observed, consistent with an adaptive response mediated by PHD inhibition. Third, despite comparable increases in haematocrit, these increases were very much smaller than those observed in response to a similar period of continuous hypoxia. Thus, the effects of PHD inhibition on ventilation, at a dose close to the maximum tolerated (and able to stimulate erythropoiesis strongly), were only very modest and clearly different from those of hypoxic exposure and PHD2 inactivation.

To pursue this further we attempted to assay for changes in the levels of different HIF-α isoforms in the carotid bodies of PHI-treated, hypoxia-treated and *PHD2*^+/−^ animals but we were not able to obtain sufficiently quantitative data for meaningful analysis. Furthermore, the different timecourse of HIF-1α*versus* HIF-2α activation by hypoxic stimulation and PHI (Holmquist-Mengelbier *et al.* 2006), together with the wholly different timecourse of inactivation in genetic *versus* pharmacological intervention (7 day exposure *versus* constitutive PHD inactivation), makes comparison by simple measurement of HIF-α levels difficult or impossible to interpret.

It is therefore possible that the differences between responses to continuous hypoxia or PHD2 heterozygosity and prolyl hydroxylase inhibition either reflect differences within the carotid body or other respiratory control centres in activation of HIF-1α and/or HIF-2α (which, in turn, can counter-regulate each other; Raval *et al.* 2005; Menrad *et al.* 2010). Alternatively, they may reflect more fundamental differences between the stimuli. For instance, unlike hypoxia, prolyl hydroxylase inhibition had no immediate effect on respiration, indicating that PHD enzyme activity is unlikely to contribute to the rapid changes in ventilation that occur in response to hypoxia. It is possible that this PHD-independent action of hypoxia is also important for the adaptive responses observed over the 7 day exposure to hypoxia.

Overall, our findings provide clear genetic evidence for a role of PHD2 in regulating ventilatory sensitivity to hypoxia. Despite this, care is required in predicting the responses to pharmacological inhibition of the PHD enzymes, though it is likely that erythropoietic stimulation by these agents can be substantially uncoupled from effects on respiratory control.

## Abbreviations

BrdU: bromodeoxyuridine
CB: carotid body
HBSS: Hanks’ buffered saline solution
HIF: hypoxia-inducible factor
HVR: hypoxic ventilatory response
PHD: prolyl hydroxylase domain enzyme
PHI: prolyl hydroxylase inhibitor
TH: tyrosine hydroxylase
VHL: von Hippel Lindau protein
