# Supplementary material for: Carotid body hyperplasia and enhanced ventilatory responses to hypoxia in mice with heterozygous deficiency of PHD2
Source: J Physiol. 2013 May 20;591(Pt 14):3565–77. doi: 10.1113/jphysiol.2012.247254 (PMC3731614; doi:10.1113/jphysiol.2012.247254)
Supplement: Supplementary file 1 [file tjp0591-3565-SD1.pdf]

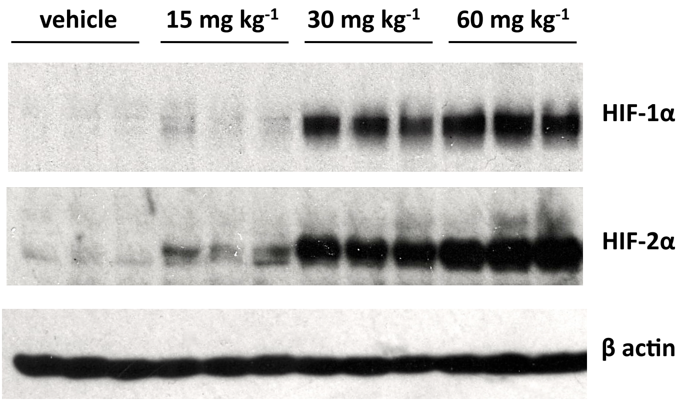

Supplementary figure 1: **Dose-dependent effect of IOX3 on HIFα protein levels in the liver.** IOX3 (or vehicle) was administered to male wild-type C57BL/6 mice by intraperitoneal injection at 15 – 60 mg kg^{-1} and livers harvested 1 hr after treatment for immunoblotting with HIF-1α and HIF-2α antibodies (Novus Biologicals; PM9) as well as with β actin. HIF-1α and HIF-2α induction was near maximal at 30 mg kg^{-1}. This dose was chosen for twice daily injections over the 7 day period as the 60 mg kg^{-1} dose was not tolerated by mice after multiple injections.

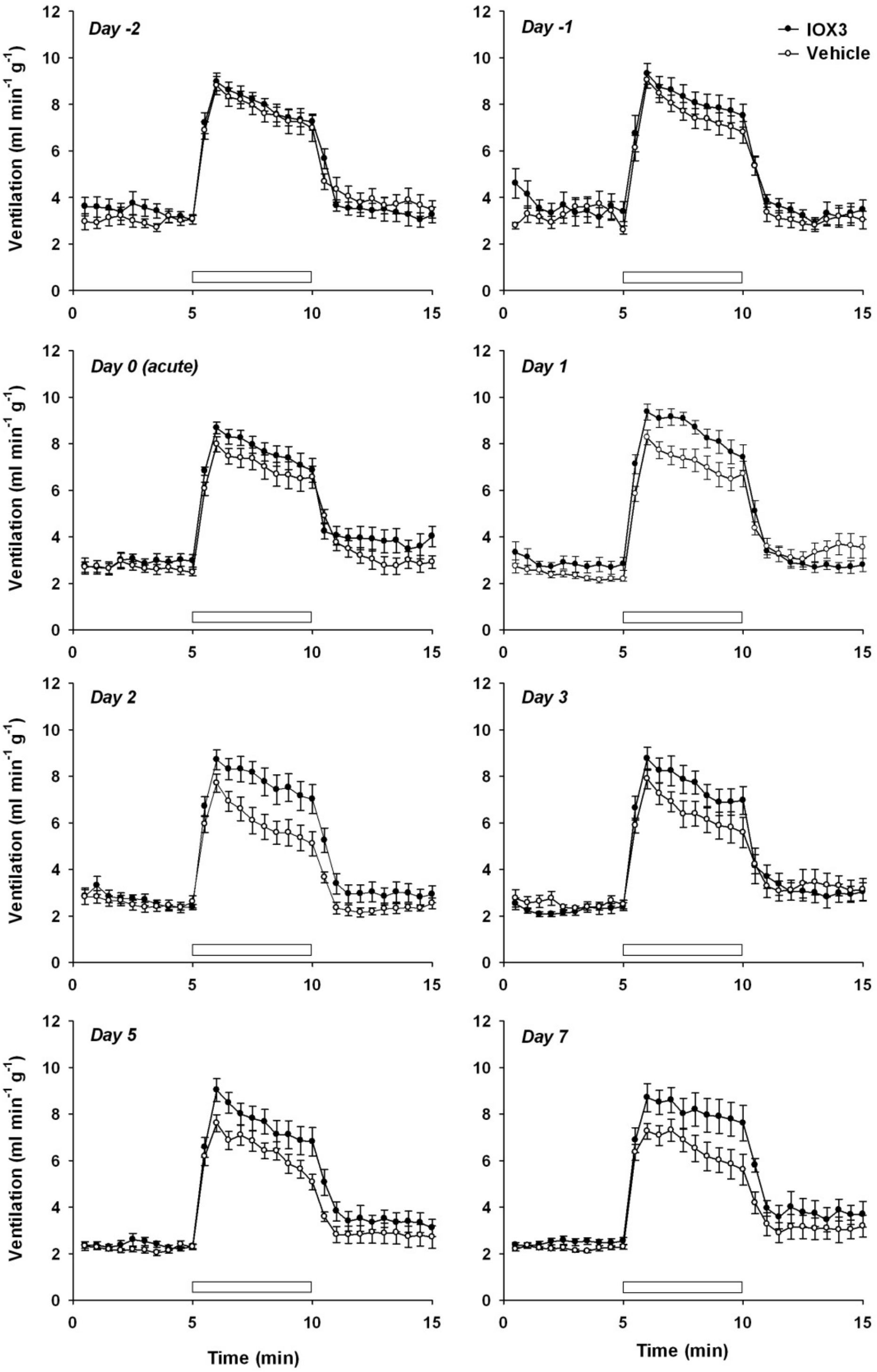

***Supplementary figure 2*: Effect of pharmacological PHD inhibition on ventilatory responses to acute hypoxia.** Baseline responses were measured before IOX3 administration (days -1 and -2), within 90 min of the first dose of IOX3 (day 0) and on days 1, 2, 3, 5 and 7 after the onset of intraperitoneal administration of IOX3 at a dose of 30 mg kg^{-1} twice daily, or vehicle control. On each day of study, mice breathed air within the chamber for at least 30 min before measurements; baseline ventilation was then measured over the next 5 min. Mice were then exposed to 5 min of 10% oxygen with 3% carbon dioxide (open bar), before returning to breathing air for a further 5 min. Values are mean ± S.E.M.; n = 11 C57BL/6 animals in each group.

Baseline ventilation

	Pre-treatment (day -1)		Post-treatment (day 7)	
	Hypoxia	Normoxia	Hypoxia	Normoxia
Minute ventilation (ml min^{-1} g^{-1})	4.3 ± 0.4	4.8 ± 0.5	5.8 ± 0.3**	3.7 ± 0.3
Tidal volume (μl g^{-1})	15.0 ± 0.8	15.8 ± 0.9	15.9 ± 0.5	14.1 ± 1.0
Respiratory rate (breaths min^{-1})	278 ± 23	253 ± 18	373 ± 17**	275 ± 17

Hypoxic ventilatory response (HVR) to 10% oxygen

	Pre-treatment HVR (day -1)		Post-treatment HVR (day 7)		Change in HVR	
	Hypoxia	Normoxia	Hypoxia	Normoxia	Hypoxia	Normoxia
Minute ventilation (ml min^{-1} g^{-1})	1.0 ± 0.5	1.0 ± 0.5	4.0 ± 0.3**	1.7 ± 0.4	3.0 ± 0.5**	0.7 ± 0.6
Tidal volume (μl g^{-1})	3.5 ± 0.9	4.0 ± 0.8	8.0 ± 0.8*	5.1 ± 0.7	4.5 ± 1.3*	1.1 ± 0.7
Respiratory rate (breaths min^{-1})	15 ± 16	29 ± 34	38 ± 22	8 ± 34	23 ± 34	-20 ± 45

Hypoxic ventilatory response (HVR) to 10% oxygen, 3% carbon dioxide

	Pre-treatment HVR (day -1)		Post-treatment HVR (day 7)		Change in HVR	
	Hypoxia	Normoxia	Hypoxia	Normoxia	Hypoxia	Normoxia
Minute ventilation (ml min^{-1} g^{-1})	5.5 ± 0.4	4.5 ± 0.7	10.8 ± 0.3**	4.1 ± 0.7	5.3 ± 0.7**	-0.3 ± 0.9
Tidal volume (μl g^{-1})	12.0 ± 1.0	11.0 ± 1.0	20.6 ± 1.1**	10.9 ± 1.7	8.6 ± 1.1**	-0.2 ± 2.0
Respiratory rate (breaths min^{-1})	84 ± 38	47 ± 35	138 ± 12*	43 ± 31	54 ± 41	-5 ± 37

***Supplementary table 1*: Effect of chronic hypoxia on hypoxic ventilatory response (HVR).** Tidal volume and respiratory rate were measured in awake animals using individual whole body plethysmographs. Baseline ventilation and HVR was measured before (day -1) and after (day 7) exposure to 7 days of 10% oxygen or room air (normoxia). On each day of study, mice breathed air within the chamber for at least 30 min before measurements; baseline ventilation was then measured over the next 5 min, before mice were exposed to 5 min of 10% oxygen, with or without the addition of 3% carbon dioxide. HVR is the difference between the relevant ventilatory parameter (minute ventilation, tidal volume or respiratory rate) during the 1 min prior to onset of hypoxia and the first 1 min of stable hypoxia (i.e. excluding the first 30 seconds after switching to hypoxic gas delivery). Values are mean ± S.E.M.; n = 8 C57BL/6 animals in each group. Comparisons between chronic hypoxia and normoxic control group were made with unpaired *t*-tests *$p<0.05$, **$p<0.01$.

Baseline ventilation

	PHD1$^{-/-}$	Wild-type	*PHD2*$^{+/-}$	Wild-type	*PHD3*$^{-/-}$	Wild-type
Minute ventilation (ml min^{-1} g^{-1})	3.6 ± 0.4	3.1 ± 0.2	5.4 ± 0.6	4.7 ± 0.4	2.9 ± 0.4	3.8 ± 0.4
Tidal volume (µl g^{-1})	12.6 ± 0.5	12.1 ± 0.7	16.2 ± 1.1	13.7 ± 0.7	13.1 ± 1.2	12.8 ± 0.6
Respiratory rate (breaths min^{-1})	300 ± 38	275 ± 15	341 ± 41	359 ± 30	227 ± 18	317 ± 44

Hypoxic ventilatory response (HVR) to 10% oxygen

	PHD1$^{-/-}$	Wild-type	*PHD2*$^{+/-}$	Wild-type	*PHD3*$^{-/-}$	Wild-type
Minute ventilation (ml min^{-1} g^{-1})	–	–	2.7 ± 0.7	1.3 ± 0.5	–	–
Tidal volume (µl g^{-1})	–	–	6.7 ± 0.8	5.3 ± 0.6	–	–
Respiratory rate (breaths min^{-1})	–	–	11 ± 35	-35 ± 38	–	–

Hypoxic ventilatory response (HVR) to 10% oxygen, 3% carbon dioxide

	PHD1$^{-/-}$	Wild-type	*PHD2*$^{+/-}$	Wild-type	*PHD3*$^{-/-}$	Wild-type
Minute ventilation (ml min^{-1} g^{-1})	4.9 ± 0.3	4.2 ± 0.4	8.4 ± 0.7**	5.0 ± 0.8	3.1 ± 0.6	3.8 ± 0.7
Tidal volume (µl g^{-1})	10.3 ± 0.6	10.6 ± 0.9	18.8 ± 1.3*	12.9 ± 2.0	6.4 ± 1.0	9.0 ± 0.9
Respiratory rate (breaths min^{-1})	140 ± 6*	58 ± 29	97 ± 45	47 ± 42	79 ± 44	36 ± 47

Supplementary table 2**: Effect of genetic *PHD* deficiency on hypoxic ventilatory response (HVR).** Tidal volume and respiratory rate were measured in awake animals using individual whole body plethysmographs. Mice breathed air within the chamber for at least 30 min before measurements; baseline ventilation was then measured over the next 5 min, before mice were exposed to 5 min of 10% oxygen, or 10% oxygen with 3% carbon dioxide. HVR is the difference between the relevant ventilatory parameter (minute ventilation, tidal volume or respiratory rate) during the 1 min prior to onset of hypoxia and the first 1 min of stable hypoxia (i.e. excluding the first 30 seconds after switching to hypoxic gas delivery). Values are mean ± S.E.M.; n = 5-7 littermate pairs in each group. *PHD2*$^{+/-}$ mice were on a pure C57BL/6 genetic background. *PHD1*$^{-/-}$ and *PHD3*$^{-/-}$ mice were on a mixed Swiss/129SvEv background. Comparisons between *PHD*-deficient and wild-type groups were made with unpaired *t*-tests *p<0.05, **p<0.01.

Baseline ventilation

	Pre-treatment (day -1)		Post-treatment (day 7)	
	Hypoxia	Normoxia	Hypoxia	Normoxia
Minute ventilation (ml min^{-1} g^{-1})	5.9 ± 0.3	5.7 ± 0.5	5.7 ± 0.2	4.8 ± 0.4
Tidal volume (μl g^{-1})	15.3 ± 0.3	14.9 ± 0.4	17.4 ± 0.1	15.2 ± 0.6**
Respiratory rate (breaths min^{-1})	400 ± 16	395 ± 33	326 ± 13	329 ± 26

Hypoxic ventilatory response (HVR) to 10% oxygen

	Pre-treatment HVR (day -1)		Post-treatment HVR (day 7)		Change in HVR	
	Hypoxia	Normoxia	Hypoxia	Normoxia	Hypoxia	Normoxia
Minute ventilation (ml min^{-1} g^{-1})	2.0 ± 1.0	2.2 ± 1.1	3.2 ± 0.8	1.7 ± 0.5	1.2 ± 0.8	-0.5 ± 0.8
Tidal volume (μl g^{-1})	6.7 ± 0.6	7.1 ± 1.0	5.2 ± 0.8	5.9 ± 0.6	-1.5 ± 0.5	-1.2 ± 0.8
Respiratory rate (breaths min^{-1})	-33 ± 58	-15 ± 53	68 ± 35	-38 ± 28*	101 ± 54	-22 ± 66

Hypoxic ventilatory response (HVR) to 10% oxygen, 3% carbon dioxide

	Pre-treatment HVR (day -1)		Post-treatment HVR (day 7)		Change in HVR	
	Hypoxia	Normoxia	Hypoxia	Normoxia	Hypoxia	Normoxia
Minute ventilation (ml min^{-1} g^{-1})	7.9 ± 0.6	8.3 ± 0.8	8.3 ± 0.6	6.6 ± 0.7	0.4 ± 0.8	-1.7 ± 1.0
Tidal volume (μl g^{-1})	17.7 ± 0.9	19.1 ± 1.1	17.0 ± 0.4	16.9 ± 1.2	-0.7 ± 1.2	-2.2 ± 1.6
Respiratory rate (breaths min^{-1})	66 ± 30	98 ± 44	67 ± 33	45 ± 39	1 ± 47	-52 ± 65

***Supplementary table 3*: Effect of chronic hypoxia on hypoxic ventilatory response (HVR) in PHD2 deficient animals.** Tidal volume and respiratory rate were measured in awake *PHD2*$^{+/-}$ animals using individual whole body plethysmographs. Baseline ventilation and HVR were measured before (day -1) and after (day 7) exposure to 7 days of 10% oxygen or room air (normoxia). On each day of study, mice breathed air within the chamber for at least 30 min before measurements; baseline ventilation was then measured over the next 5 min, before mice were exposed to 5 min of 10% oxygen, with or without the addition of 3% carbon dioxide. HVR is the difference between the relevant ventilatory parameter (minute ventilation, tidal volume or respiratory rate) during the 1 min prior to onset of hypoxia and the first 1 min of stable hypoxia (i.e. excluding the first 30 seconds after switching to hypoxic gas delivery). Values are mean ± S.E.M.; n = 5 C57BL/6 animals in each group. Comparisons between chronic hypoxia and normoxic control group were made with unpaired *t*-tests *$p<0.05$, **$p<0.01$.

Baseline ventilation

	Pre-treatment (day -1)		Post-treatment (day 0)		Post-treatment (day 7)	
	IOX3	Vehicle	IOX3	Vehicle	IOX3	Vehicle
Minute ventilation (ml min^{-1} g^{-1})	4.5 ± 0.5	3.8 ± 0.3	3.6 ± 0.3	2.8 ± 0.2	3.0 ± 0.3	3.0 ± 0.3
Tidal volume (µl g^{-1})	12.7 ± 0.5	12.5 ± 0.5	12.2 ± 0.3	11.5 ± 0.5	12.0 ± 0.4	11.4 ± 0.3
Respiratory rate (breaths min^{-1})	358 ± 37	317 ± 23	302 ± 23	251 ± 20	252 ± 21	271 ± 26

Day 0: Hypoxic ventilatory response (HVR) to 10% oxygen, 3% carbon dioxide

	Pre-treatment HVR (day -1)		Post-treatment HVR (day 0)		Change in HVR	
	IOX3	Vehicle	IOX3	Vehicle	IOX3	Vehicle
Minute ventilation (ml min^{-1} g^{-1})	5.5 ± 0.4	5.7 ± 0.3	5.5 ± 0.4	5.2 ± 0.3	0.0 ± 0.7	-0.5 ± 0.4
Tidal volume (µl g^{-1})	13.5 ± 0.9	13.5 ± 1.0	13.4 ± 1.1	12.3 ± 1.2	-0.1 ± 1.1	-1.2 ± 1.2
Respiratory rate (breaths min^{-1})	68 ± 30	96 ± 15	83 ± 22	100 ± 19	15 ± 45	5 ± 18

Day 7: Hypoxic ventilatory response (HVR) to 10% oxygen, 3% carbon dioxide

	Pre-treatment HVR (day -1)		Post-treatment HVR (day 7)		Change in HVR	
	IOX3	Vehicle	IOX3	Vehicle	IOX3	Vehicle
Minute ventilation (ml min^{-1} g^{-1})	5.5 ± 0.4	5.7 ± 0.3	6.1 ± 0.6	4.9 ± 0.3	0.6 ± 0.5*	-0.9 ± 0.3
Tidal volume (µl g^{-1})	13.5 ± 0.9	13.5 ± 1.0	13.5 ± 1.1	11.2 ± 0.9	0.0 ± 0.7*	-2.3 ± 0.7
Respiratory rate (breaths min^{-1})	68 ± 30	96 ± 15	129 ± 14	115 ± 9	61 ± 35	19 ± 15

***Supplementary table 4*: Effect of pharmacological PHD inhibition on hypoxic ventilatory response (HVR).** Tidal volume and respiratory rate were measured in awake animals using individual whole body plethysmographs. Baseline ventilation and HVR were measured before administration of the PHD inhibitor IOX3 (day -1), within 90 min of the first dose of IOX3 (day 0) and after 7 days of intraperitoneal IOX3 at a dose of 30 mg kg^{-1} twice daily, or vehicle control. On each day of study, mice breathed air within the chamber for at least 30 min before measurements; baseline ventilation was then measured over the next 5 min before mice were exposed to 5 min of 10% oxygen with 3% carbon dioxide. HVR is the difference between the relevant ventilatory parameter (minute ventilation, tidal volume or respiratory rate) during the 1 min prior to onset of hypoxia and the first 1 min of stable hypoxia (i.e. excluding the first 30 seconds after the switch to hypoxic gas delivery). Values are mean ± S.E.M.; n = 11 C57BL/6 animals in each group. Comparisons between IOX3 and vehicle-treated groups were made with unpaired *t*-tests *$p<0.05$, **$p<0.01$.
